# Direct targeting of *C9ORF72* repeat RNA with fluorinated antisense oligonucleotides

**DOI:** 10.1093/nar/gkag343

**Published:** 2026-04-25

**Authors:** Halle M Barber, Mansi A Parasrampuria, Jerónimo Jurado-Arjona, Andrea Gamir-Morralla, Benedikt Berninger, Carlos González, Keith T Gagnon, Masad J Damha, Miguel Garavís

**Affiliations:** Department of Chemistry, McGill University, Montreal, QC H3A 0B8, Canada; Biochemistry and Molecular Biology, Wake Forest University School of Medicine, Winston-Salem, NC 27101, United States; Division of Biochemistry and Molecular Biology, Southern Illinois University School of Medicine, Carbondale, IL 62901, United States; Centre for Developmental Neurobiology, Institute of Psychiatry, Psychology & Neuroscience, King’s College London, London SE1 1UL, United Kingdom; Institute of Physiological Chemistry, University Medical Center Johannes Gutenberg University, Mainz 55128, Germany; Centre for Developmental Neurobiology, Institute of Psychiatry, Psychology & Neuroscience, King’s College London, London SE1 1UL, United Kingdom; Institute of Physiological Chemistry, University Medical Center Johannes Gutenberg University, Mainz 55128, Germany; Centre for Developmental Neurobiology, Institute of Psychiatry, Psychology & Neuroscience, King’s College London, London SE1 1UL, United Kingdom; Institute of Physiological Chemistry, University Medical Center Johannes Gutenberg University, Mainz 55128, Germany; MRC Centre for Neurodevelopmental Disorders, Institute of Psychiatry, Psychology & Neuroscience, King’s College London, London SE1 1UL, United Kingdom; Focus Program Translational Neuroscience, Johannes Gutenberg University, Mainz 55131, Germany; Instituto de Química Física ‘Blas Cabrera’, IQF-CSIC, Madrid 28006, Spain; Biochemistry and Molecular Biology, Wake Forest University School of Medicine, Winston-Salem, NC 27101, United States; Division of Biochemistry and Molecular Biology, Southern Illinois University School of Medicine, Carbondale, IL 62901, United States; Department of Chemistry, McGill University, Montreal, QC H3A 0B8, Canada; Instituto de Química Física ‘Blas Cabrera’, IQF-CSIC, Madrid 28006, Spain

## Abstract

Hexanucleotide repeat expansions in the *C9ORF72* gene are the most common genetic cause of amyotrophic lateral sclerosis and frontotemporal dementia. These expansions give rise to pathogenic sense and antisense repeat RNAs that form nuclear foci and undergo repeat-associated non-AUG translation, producing dipeptide repeat proteins with cellular toxicity. Directly targeting the causative repeat RNAs with antisense oligonucleotides represents a promising therapeutic strategy. One barrier to further development is the propensity of this G-rich repeat-containing RNA target to form stable secondary structures, which may hinder efficient hybridization. In this study, we designed a panel of fluorine-modified ASOs that target the sense repeat expansions. We identified C-rich F-ASO gapmers that reduced translation from sense repeat RNAs in a cell-based reporter assay and lowered the RNA foci burden in patient-derived cells. Structural analyses *in vitro* revealed that the 2′F-RNA gapmer formed a stable hairpin structure. Our results demonstrate that structural properties of fluorine modifications can be leveraged for effective binding of repeat RNA and highlight the potential for F-ASOs to serve as therapeutic tools when targeting toxic repeat RNAs in *C9ORF72*-mediated FTD/ALS and other repeat expansion diseases.

## Graphical Abstract

**Graphical Abstract:**
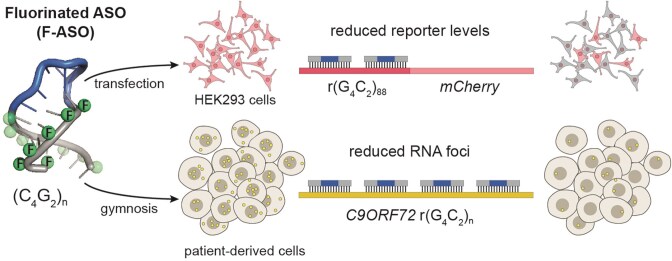

## Introduction

The formation of an antiparallel double-stranded helix between an endogenous RNA target and an antisense oligonucleotide (ASO) is the fundamental process that activates the mechanisms on which antisense therapy is based [1]. The use of antisense therapeutics has become a reference strategy to combat disease caused by genetic mutations. To date, 13 ASOs have been approved by the Food and Drug Administration (FDA), the European Medicines Agency (EMA), or both to treat different diseases [2, 3]. In the last decade, several reports have focused on the use of antisense-based therapeutics for frontotemporal dementia (FTD) and amyotrophic lateral sclerosis (ALS) associated with a hexanucleotide repeat expansion within the *C9ORF72* gene [4–13], referred to collectively as C9FTD/ALS. The expansion of the sequence 5′-GGGGCC-3′/3′-CCCCGG-5′ located in the first intron of the gene is the most frequent genetic cause of familial ALS and FTD [14, 15]. Transcription leads to production of sense (G_4_C_2_) and antisense (C_4_G_2_) repeat-containing RNAs that induce a cascade of molecular disease mechanisms. On one hand, mutated RNAs accumulate as aberrant nuclear foci [14, 16] that can sequester RNA-binding proteins, potentially leading to a loss of function. On the other hand, a gain-of-function mechanism has been proposed through repeat-associated non-AUG (RAN) translation, giving rise to dipeptide repeat proteins (DPRs), which contribute to toxicity and cell damage [17–21].

The substantial number of repeats occurring in the *C9ORF72* gene and other genes associated with repeat-expansion disorders offers a potential window of therapeutic opportunity. Although the repeat sequence itself may be common, its expansion is usually unique, which may enable specific targeting. In addition, binding of an ASO to the expanded repeat sequence may prevent some pathogenic mechanisms, such as the aberrant binding of proteins, repeat-induced RNA aggregation, and translation into protein. Importantly, targeting the expanded repeat region might help in preventing C9ORF72 haploinsufficiency [22]. Several ASOs and siRNAs have previously been explored to target repeat RNAs associated with repeat-expansion disorders [23–26], including those arising from the *C9ORF72* hexanucleotide expansion [4, 8, 9, 27]. Notably, two ASOs targeting non-repeat regions progressed to clinical trials but were discontinued after phase 1 [28, 29]. One challenge is the potential self-folding of the ASO and target RNA into stable structures such as R-loops, hairpins, G-quadruplexes, or i-motifs [30], which are particularly favored in GC-rich repeat-containing sequences. These may hinder the formation of the ASO:RNA duplex required for antisense activity. Therefore, characterizing ASO structure and its impact on binding to complementary RNA is an important step toward using ASOs to target GC-rich repeat sequences.

Fluorine substitution at the 2′ position of the ribose sugar (2′F-RNA) has been extensively used in oligonucleotide therapeutics, including those approved for use in the clinic [31, 32]. Additionally, oligonucleotides containing 2′-fluoroarabinose sugars (2′F-ANA) have unique properties that make them very suitable ASOs [33–37]. The presence of fluorine in the sugar has noteworthy consequences such as the stabilization of non-canonical structures and ASO:RNA duplexes [33, 38–40]. Therefore, fluorine modifications may help to overcome some of the structure-related limitations of ASOs in repeat expansion diseases such as ALS and FTD.

Herein, we have synthesized a series of ASOs containing fluorine modifications (F-ASOs) that target the *C9ORF72* sense repeat sequences. We show that some F-ASOs effectively reduce phenotypic features in cellular models harboring the repeat expansion. Using biophysical methods, we demonstrate that the most active F-ASOs fold into stable secondary structures at near physiological conditions. However, these do not preclude their ability to hybridize with fully complementary repeat RNAs that also possess secondary structure.

## Materials and methods

### Chemical synthesis of antisense oligonucleotides

All unmodified DNA and RNA oligonucleotides were purchased from Integrated DNA Technologies (IDT). All chemically modified oligonucleotides were synthesized on either an ABI 3400 DNA synthesizer (Applied Biosystems) or a MerMade 12 oligonucleotide synthesizer (LGC Biosearch Technologies) via solid-phase oligonucleotide synthesis (Supplementary Table S1). Phosphoramidites (ChemGenes) were prepared as 0.1 M solutions in acetonitrile (ACN), except for LNA 5-methyl cytidine, which was prepared in ACN:dichloromethane (DCM) (1:1, v/v). 500 Å Unylinker CPG (ChemGenes) was used as a solid support on a 1 µmol scale. Detritylation occurred at the beginning of each cycle and at the end of the synthesis to remove the dimethoxytrityl groups using 3% trichloroacetic acid in DCM. The phosphoramidites were activated for coupling with 0.25 M 5-ethylthio-1H-tetrazole in ACN. Coupling times were 3 min for DNA, 2′F-RNA, and LNA, and 6 min for 2′F-ANA (Glen Research). Sequences that failed to couple were capped with Cap A [acetic anhydride in pyridine/tetrahydrofuran (THF)] and Cap B (16% *N*-methylimidazole in THF). The sequences were either treated with 0.1 M iodine in pyridine/water/THF to create phosphodiester linkages or treated with 0.1 M 3-[(dimethylamino-methylidene)amino]-3H-1,2,4-dithiazole-3-thione in pyridine/ACN to create phosphorothioate linkages. Following synthesis, the oligonucleotides were deprotected using cold 28%–30% aqueous ammonia at room temperature (RT) for 48 h and filtered. The crude oligonucleotides were purified on a Waters 1525 anion-exchange HPLC using a Source 15Q resin column (11.5 cm × 3 cm) with a flow rate of 10 ml/min at 60°C using a gradient of 0%–100% buffer B over 50 min. Buffer A consisted of 10 mM sodium acetate (NaOAc) pH 5.5 in 25% ACN in water, and buffer B consisted of 0.5 M LiClO_4_ and 10 mM NaOAc pH 5.5 in 25% ACN in water. The purified oligonucleotides were desalted using Gel Pak 2.5 size-exclusion columns (Glen Research) and characterized using electrospray ionization LC-MS. Extinction coefficients were calculated using the IDT OligoAnalyzer Tool (Integrated DNA Technologies).

### Culturing reporter cells

HEK293 Tet-ON 3G cells were engineered to stably and inducibly express poly-GP variants of various lengths, encoded by uninterrupted (G_4_C_2_)_1_ or (G_4_C_2_)_88_ repeats fused to an mCherry reporter. The GGGGCC repeats were initially cloned via recursive directional ligation, following a previously published method [41]. These constructs were packaged commercially by Vector Builder and transduced into HEK Tet-ON 3G cells according to the manufacturer’s recommendations. These cells were cultured in 1× Minimal Essential Medium (MEM; Corning), supplemented with 5% fetal bovine serum (tetracycline-free) (FBS; TaKaRa) and 1× non-essential amino acids (NEAA) (100×, Gibco), at 37°C in a 5% CO_2_ environment.

### Culturing patient-derived neural stem cells

The patient (FTD26) induced pluripotent stem cells (iPSCs) were a kind gift from Dr Fen-Biao Gao at the University of Massachusetts Medical School [42]. These iPSC patient cell lines were differentiated into the patient neural stem cells (NSCs) following the commercially available, 7-day serum-free approach [43]. For NSC culture, tissue culture flasks, 35-mm microscopic dishes, and 96-well plates were coated with Geltrex (Gibco), a reduced growth factor basement membrane matrix, diluted 1:100 in DMEM/F-12, followed by storage at 4°C until use. The NSC growth media StemPro NSC SFM (Gibco) was prepared by aseptically combining Knockout DMEM/F-12 (500 ml) (Gibco), bFGF Basic Recombinant Human (10 µg) (Gibco), EGF Basic Recombinant Human (10 µg) (Gibco), StemPro Neural Supplement (10 ml) (Gibco), and Glutamax-I Supplement (1×) (Gibco). The patient-derived NSCs were cultured and maintained in humidified incubator at 37°C with 5% CO_2_ and passaged every 3–4 days using TrypLE Express Enzyme (Gibco) upon reaching ∼80% confluency.

### Culturing human induced pluripotent stem cells

Human induced pluripotent stem cells (hiPSCs) derived from an ALS patient (UCLi004-A/Rci173) were obtained from EBiSC. Cells were cultured on matrigel-coated six-well plates using mTeSR Plus medium (StemCell Technologies) supplemented with 100 units/ml PenStrep (Gibco) in a humidified incubator at 37°C, 5% CO_2_. For passaging, cells were washed with DPBS (no calcium, no magnesium; Gibco), incubated with 0.5 mM ethylenediaminetetraacetic acid (EDTA) (Invitrogen) for 3 min at RT, and gently detached and split in mTeSR Plus media. For subsequent RNA-FISH experiments, cells were grown on matrigel-coated 13-mm glass coverslips. Cells were treated with ASOs at 10, 30, and 50 nM for 4 days, with medium and treatment refreshed at day 2.

### ASO transfection

To investigate the potential of GGGGCC-targeting ASOs as a therapeutic approach in repeat-associated neurodegeneration, we tested the ASOs in reporter cells and patient-derived NSCs. The positive control, MOE_Ctrl, was purchased from IDT. Cells were seeded using the previously mentioned culturing conditions. Transfections were carried out using Lipofectamine RNAiMAX (Invitrogen) according to the manufacturer’s protocol, unless otherwise noted. ASOs were diluted in Opti-MEM Reduced Serum Medium (Gibco) in the presence of RNAiMAX, using a 1:1 serial dilution series to generate the desired final concentrations. Transfected cells were incubated at 37°C and 5% CO_2_. Phenotypic analysis was performed at the indicated time points post-transfection.

### Flow cytometry

Flow cytometry was performed to quantify mCherry expression 96 h post-transfection in reporter cells treated with the ASOs. Cells were harvested using 1× Trypsin–EDTA (Gibco), centrifuged at 300 × *g* for 5 min, and resuspended in 200 μl of phosphate-buffered saline (PBS) to obtain a single-cell suspension. mCherry expression and transfection efficiency were analyzed using an Attune NxT Flow Cytometer with Autosampler (Thermo Fisher Scientific), equipped with a 488 nm blue laser and a 561 nm yellow laser for mCherry excitation. Emission was detected at 620 nm. A maximum of 40 000 events per sample were collected at a flow rate of 140 μl/min. Cells were first gated on forward scatter area (FSC-A) versus side scatter area (SSC-A) to exclude debris, followed by gating for singlets using FSC height (FSC-H) versus FSC-A. mCherry-positive cells were detected in the YL2-A channel, and the mean fluorescence intensity (MFI) of RFP was used to assess expression levels (Supplementary Figs S1 and S2). Quadrant gates for mCherry positivity were set based on untreated, doxycycline-negative control cells. All experiments were performed in quadruplicate (*n* = 4). Statistical analyses, including the calculation of mean values and standard deviation (SD), were conducted using GraphPad Prism (GraphPad Software, version 10.3.0).

### Cell viability assay

To determine cell viability and cytotoxicity of ASOs in patient-derived NSCs and human induced pluripotent stem cells (hiPSCs), a colorimetric cell proliferation assay (MTS assay) was performed using the CellTiter 96 AQueous One Solution Cell Proliferation Assay kit (Promega), following the manufacturer’s guidelines.

For NSCs, cells were seeded in 96-well plates at a 30 000 cells per well seeding density and incubated for 24 h at 37°C in a humidified incubator with 5% CO₂. Cells were then transiently transfected with C-rich ASOs at a final concentration of 20 nM and incubated for an additional 48 h under the same conditions. Following treatment, the culture medium was removed, and 100 µl per well of fresh 1× Minimal Essential Medium (MEM, without glutamine and phenol red; Gibco) containing the CellTiter 96 AQueous One Solution reagent was added. Plates were incubated for 2 h at 37°C in the dark. Absorbance was measured at 490 nm using an Agilent BioTek 96-well plate reader. Background absorbance from “no-cell” control wells was subtracted from each experimental well to obtain corrected absorbance values. The data were analyzed and plotted using GraphPad Prism software (version 10.3.0), and results were presented as mean ± SD from at least four independent replicates.

For hiPSCs, 6000 cells per well were seeded in 96-well plates and incubated at 37°C in a humidified incubator with 5% CO₂. After 24 h, cells were treated with ASOs at 50 nM for 4 days, with medium and treatment refreshed on day 2. After the treatment, the culture medium was removed and 100 µl per well of fresh mTeSR Plus Medium together with CellTiter 96 AQueous One Solution reagent was added. Plates were incubated for 2 h at 37°C in the dark. Absorbance was measured at 450 nm using a Thermo Fisher MultiSkan FC 96-well plate reader. Background absorbance from “no-cell” control wells was subtracted from each experimental well to obtain corrected absorbance values. The data were analyzed from two technical replicates per experiment and plotted using GraphPad Prism software (version 10.3.0), and results were presented as mean ± standard error of the mean (SEM) from three independent experiments.

### Fluorescent *in situ* hybridization in patient-derived NSCs

RNA fluorescent in situ hybridization (FISH) was performed to visualize GGGGCC repeat-containing RNA foci in patient-derived NSCs. A Cy5-labeled DNA probe complementary to the sense RNA foci sequence was used: TYE665(C_4_G_2_)_4_, purchased from IDT. The probe was resuspended in 1× probe buffer consisting of 1× TE, 70% formamide, and 20% DMSO. Prior to hybridization, the probe was denatured at 95°C for 2–3 min and snap-cooled on ice. For FISH, 250 000 patient-derived NSCs were seeded per well in 35 mm glass-bottom imaging dishes. After 72 h of ASO transfection, cells were washed with 1× PBS and fixed with 3.7% formaldehyde in 1× PBS for 10 min at RT. Fixed cells were washed twice for 5 min with 1× PBS and then permeabilized in 70% ethanol for 4 h at RT or overnight at 4°C.

Cells were equilibrated in wash buffer (40% formamide, 2× saline-sodium citrate [SSC]) for 5 min, followed by pre-hybridization in hybridization buffer (100 mg/ml dextran sulfate, 40% formamide, 2× SSC) for 30 min at 45°C. Hybridization was performed in the same buffer containing the TYE665(C_4_G_2_)_4_ probe at a final concentration of 80 nM, incubated at 45°C for 6–8 h in a light-protected, humidified chamber.

Post-hybridization, cells were washed four times for 1 h each in wash buffer at 45°C, followed by a final 5-min wash with 1× PBS at RT. Nuclei were counterstained using Vectashield antifade mounting medium with DAPI (Vector Labs, H-1200), and coverslips were sealed. Imaging was performed using a Leica DMi8 confocal microscope equipped with a 63× oil immersion objective. Images were acquired with Leica LAS X software. For each condition, two biological replicates were analyzed, with 8–10 fields of view captured per replicate. On average, 200–250 nuclei were manually counted based on DAPI staining. Sense RNA foci were visualized in the Cy5 channel and quantified using two parameters: (i) the number of foci per cell, and (ii) the percentage of cells containing foci. Data were analyzed using GraphPad Prism software, and results were reported as mean ± SEM.

### FISH in patient-derived hiPSCs

DNA oligonucleotide fluorescent probe (G_2_C_4_)_3_Alexa647 complementary to the expansion repeats of *C9ORF72* gene was purchased from IDT. Cells grown on matrigel-coated coverslips were rinsed once with 1× PBS and fixed with 4% formaldehyde (Electron Microscopy) for 15 min at RT. After fixation, cells were washed three times with 1× PBS for 5 min each, permeabilized with 70% ethanol for 1 h at RT, and stored at 4°C in 70% ethanol. On the day of the FISH staining, cells were rinsed for 2 min in 2× SSC buffer (Thermo Fisher Scientific) with 15% formamide. Coverslips were placed in a humid chamber and incubated with the fluorescent-labeled probe diluted at 125 nM in hybridization buffer (2× SSC, 15% formamide and 10% dextran sulfate) overnight at 42°C. Next day, the samples were washed with 2× SSC buffer and 15% formamide for 30 min at 42°C and 1× SSC buffer for 15 min at RT. Afterwards, coverslips were rinsed in 1× PBS for 1 min and stained with DAPI in 1× PBS for 3 min at RT. Finally, samples were briefly rinsed in 1× PBS and mounted onto microscope slides using Mowiol (Polyscience). Z-stack images were taken with 1 μm interval between planes using a ZEISS 800 confocal microscope with Plan-Apochromat 63×/1.4 oil immersion objective. A double-blinded quantification of the number of RNA foci per cell was performed, based on three independent biological experiments, each analyzed in two technical replicates.

Cells counted: Control cell line from healthy donor, 913; UNT, 579; ASO3 10 nM, 436; ASO3 30 nM, 463; ASO3 50 nM, 568; ASO3_Scr 10 nM, 519; ASO3_Scr 30 nM, 493; ASO3_Scr 50 nM, 474; ASO4 10 nM, 586; ASO4 30 nM, 579; ASO4 50 nM, 466; ASO4_Scr 10 nM, 558; ASO4_Scr 30 nM, 432; ASO4_Scr 50 nM, 603; MOE_Ctrl 10 nM, 448; MOE_Ctrl 30 nM, 444; MOE_Ctrl 50 nM, 412.

### Thermal denaturation of the ASO:RNA duplexes

Experiments were performed on either a Varian Cary 100 Bio UV-Visible Spectrophotometer equipped with a Cary Temperature Controller or an Agilent Cary 3500 UV-Visible Spectrophotometer using a 1-cm path length cuvette. Duplexes were prepared at a concentration of 2 µM duplex in 10 mM sodium phosphate buffer pH 7.4 with a final sample volume of 500 µl. Samples were annealed by heating to 95°C for 10 min, slowly cooling to RT over 3 h, and then incubating at 4°C overnight. Absorbance values were measured at 260 nm (and 295 nm in the case of RNA21) over the range of 20 to 95°C at a ramp rate of 0.5°C/min with a data collection interval of 0.5°C. The maximum value of the first derivative curves was used to determine the melting temperature (T_m_) values. The average T_m_ from the three replicates with the corresponding standard deviation is reported along with the normalized absorbance spectra.

### NMR experiments and modeling

Oligonucleotide samples for NMR experiments were prepared at a final concentration of ~0.2 mM and in a volume of 200 µl of buffer (H_2_O/D_2_O 9:1) containing 10 mM sodium phosphate pH 7.4, 110 mM KCl, and 2 mM MgCl_2_. NMR spectra were acquired at 20°C and 37°C on Bruker Avance spectrometers equipped with cryoprobes and operating at either 600 or 800 MHz. Two-dimensional spectra of ASO4_PO were recorded at 20°C under the same oligonucleotide concentration and buffer conditions mentioned above. TOCSY spectrum was acquired with the standard MLEV17 spinlock sequence and with an 80 ms mixing time. NOESY spectrum was acquired with a mixing time of 150 ms. In both 1D and 2D spectra, water suppression was achieved by including an excitation sculpting scheme [44] in the pulse sequence prior to acquisition. G:C base pairs were detected by analyzing guanine imino protons chemical shifts and their cross-peaks with cytosine amino protons. The computer package Vfold pipeline [45] was used to model the three-dimensional structure of ASO4_PO on the basis of the G:C base pairs experimentally detected. PyMOL (Shrödinger, LLC) was used to represent the model.

### Circular dichroism of the duplexed and single-stranded oligonucleotides

Experiments were performed on a Chirascan VX spectrophotometer (Applied Photophysics) using a 1 mm path length cuvette. Samples were prepared at a concentration of 20 µM of either duplex or single-stranded ASO or RNA with a final sample volume of 200 µl with buffer containing 10 mM sodium phosphate pH 7.4, 110 mM KCl, and 2 mM MgCl_2_. The slow-annealing conditions were the same as for thermal denaturation experiments. Ellipticity spectra were recorded at RT over the range of 320 to 200 nm with a bandwidth of 1 nm, a sampling rate of 0.5 s/point, and a data collection interval of 1 nm. Three acquisitions were obtained for each oligonucleotide to create an average spectrum, from which the background spectrum was subtracted. The final reported spectra were smoothed using Chirascan software.

### Native gel shift mobility assay

Four stock solutions of the samples were prepared in 1× MOPS pH 7.4, 10 mM sodium phosphate pH 7.4, 110 mM KCl, and 2 mM MgCl_2_ buffer: 50 µM ASO:RNA duplex annealed, 100 µM ASO non-annealed, 100 µM RNA non-annealed, and 100 µM RNA annealed. The following 20 µM samples were then prepared: (i) duplex annealed, (ii) ASO non-annealed, (iii) RNA non-annealed, (iv–v) ASO non-annealed + RNA non-annealed, and (vi–vii) ASO non-annealed + RNA annealed. Samples (i), (ii), (iii), (iv), and (vi) were incubated at 20°C for 16 h, and samples (v) and (vii) were incubated at 37°C for 16 h. The samples were then run on a native gel with 20% acrylamide and 1× MOPS pH 7.4, stained with Stains-All, and visualized using a Bio-Rad Gel Doc XR+ gel scanner with Image Lab Software.

## Results

### F-ASOs reduce translation of a mRNA carrying G_4_C_2_ repeats

We synthesized a series of ASOs targeting the repeated sense RNA of the *C9ORF72* gene and evaluated their efficacy through a cell-based screen using reporter cells. To assess the functional activity of the ASOs, we performed flow cytometry on HEK293 Tet-ON 3G cells stably expressing either 1 or 88 G_4_C_2_ repeats upstream of an *mCherry* reporter gene. Cells were treated with each ASO at a final concentration of 30 nM and the expression levels of mCherry were quantified using flow cytometry (Supplementary Figs S1 and S2). This reporter assay was used as an initial evaluation of ASO activity and selectivity between the (G_4_C_2_)_1_ and (G_4_C_2_)_88_ cell lines, rather than for calculation of maximal potency.

**Figure 1.**
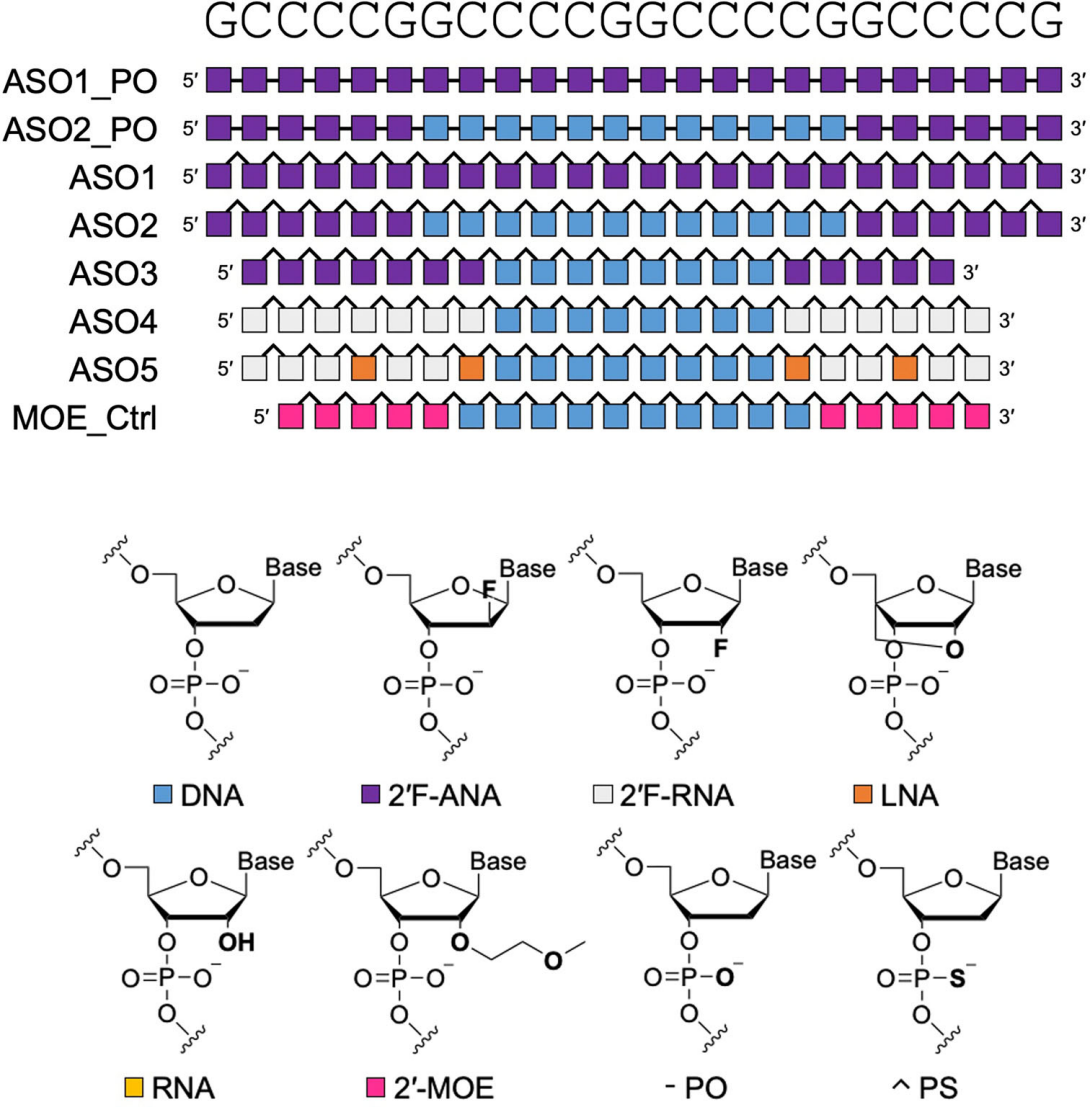
Sequences and chemical architectures of the ASOs used in this study. Structures and color/symbol codes are displayed below.

**Figure 2.**
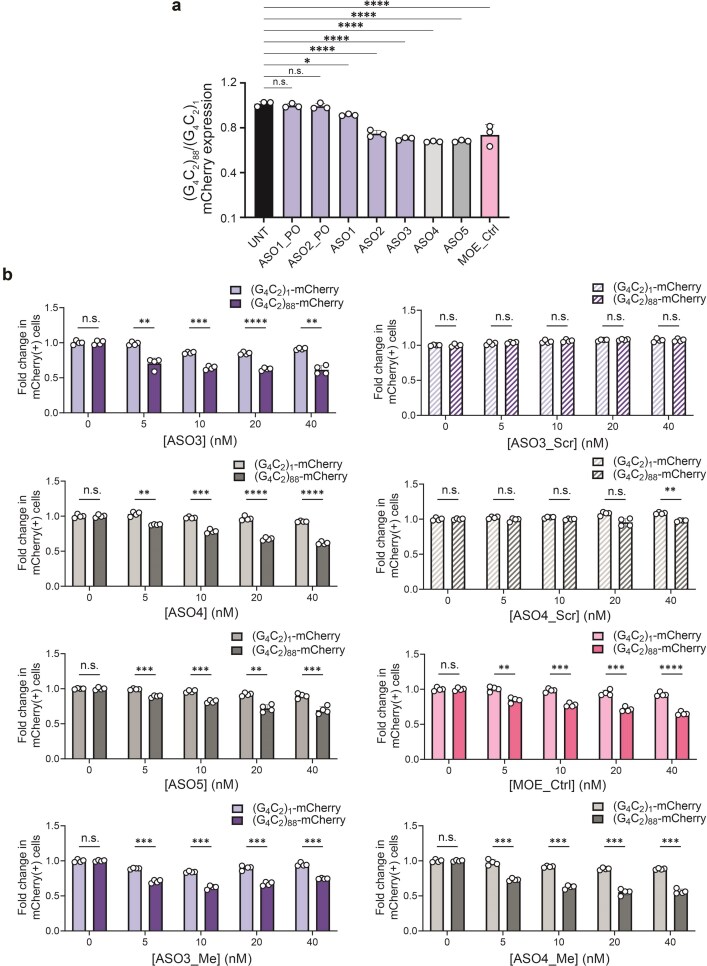
Flow cytometry-based reporter assay using HEK293 cells stably expressing either 1 or 88 G_4_C_2_ repeats in the poly-GP reading frame upstream of an *mCherry*-coding sequence. (**a**) Bar plot showing the ratio of the mCherry expression in untreated or treated cells with 88 repeats over 1 repeat. Cells were treated by transfection with 30 nM total ASO concentration (*n* = 3). Bars represent the mean ± SD from three independent experiments. Statistical significance was determined using one-way ANOVA with Dunnett’s correction. *P-value <.032; ****P-value <.0001; n.s., not significant. (**b**) Dose-response plots of mCherry expression in cells independently expressing one (light) and 88 (dark) repeats at different total ASO concentrations (*n* = 4). Values were normalized to untreated cells. Statistical significance was determined using multiple unpaired t-tests. **P-value <.0021; ***P-value <.0002; ****P-value <.0001; n.s., not significant.

We first designed 24-nucleotide-long (24-mer) full 2′F-ANA (ASO1_PO and ASO1) and 6-12-6 2′F-ANA gapmers (ASO2_PO and ASO2) having either a phosphate (PO) or phosphorothioate (PS) backbone (Fig. 1). PO-backbone ASOs were primarily included as negative controls to assess the requirement for phosphorothioate linkages in cellular activity. As expected, flow cytometry analysis revealed that PO-backbone ASOs (ASO1_PO and ASO2_PO) did not significantly reduce expression of (G_4_C_2_)_88_ relative to (G_4_C_2_)_1_ expression while the PS full 2′F-ANA ASO1 only showed a modest effect (Fig. 2a). In contrast, the PS gapmer ASO2 showed a significant reduction of (G_4_C_2_)_88_-mCherry expression. Therefore, we tested the shorter PS gapmer ASO3 (Fig. 1), which appeared to offer a slight improvement over the 24-mer analogue ASO2 (Fig. 2a).

To explore the activity of gapmers with other fluorine-based modifications, we tested the PS 2′F-RNA gapmers ASO4 and ASO5, the latter having four locked nucleic acid (LNA) nucleotides interspersed along its wings for increased binding affinity [46] (Fig. 1). Additionally, a 2′F-RNA nucleotide was added at the 3′ end of each of these sequences to ensure stable base pairing at this terminus and to determine whether this resulted in any significant difference in activity. These designs resulted in knockdown of (G_4_C_2_)_88_-mCherry similar to that of ASO3 (Fig. 2a). The effect of ASO2 through ASO5 was similar to that of a standard 5-10-5 gapmer design containing 2′-methoxyethyl (MOE) wings and a DNA gap with complete PS modification (MOE_Ctrl) (Fig. 1) used here as a positive control.

To confirm these observations, we performed a titration of several F-ASOs and our MOE_Ctrl control (Fig. 2b). Increasing concentrations of ASO3, ASO4, and ASO5 led to a selective reduction of (G_4_C_2_)_88_-mCherry expression, with no significant effect on (G_4_C_2_)_1_-mCherry. These F-ASOs performed similarly to the MOE_Ctrl control at all concentrations. We also tested two scrambled negative controls (ASO3_Scr and ASO4_Scr), having the same nucleotide composition as ASO3 and ASO4 but with different sequences. As expected, ASO3_Scr and ASO4_Scr have a negligible effect on the expression of mCherry regardless of concentration. Lastly, cytosine-guanine (CpG) motifs in ASOs are known to induce an immune response via interaction with Toll-like receptor 9 (TLR9) [47]. Methylation of deoxycytidine at the position 5 (m^5^C) is the most frequent strategy to counteract such undesired immune response. Given that the F-ASOs contain a DNA CpG motif in their sequences, we synthesized and tested methylated analogues of ASO3 and ASO4. We found that the methylated ASO3_Me and ASO4_Me reduced expression of mCherry to a similar extent as their unmethylated analogues.

### F-ASO gapmers reduce disease-associated repeat RNA foci in patient-derived NSCs

To determine whether the F-ASOs can reduce G_4_C_2_ disease-associated RNA foci, we tested them in NSCs derived from an FTD patient carrying the *C9ORF72* repeat expansion [38]. The number of sense (G_4_C_2_) repeat RNA nuclear foci was detected by FISH (Supplementary Figs S3 and S4). FISH data confirmed that a PS backbone is required for activity of both 24-mer full 2′F-ANA (ASO1_PO versus ASO1) and 2′F-ANA gapmers (ASO2_PO versus ASO2) (Supplementary Fig. S3). Shorter 2′F-ANA and 2′F-RNA gapmer versions (ASO3, ASO4, and ASO5) showed slightly improved efficacy in reducing both the number of foci per cell and the percentage of cells containing sense repeat RNA foci (Supplementary Fig. S3). MOE_Ctrl also showed comparable efficiency to the most active F-ASOs at reducing RNA foci. Treatment of cells with scrambled negative controls ASO3_Scr and ASO4_Scr caused some idiosyncratic reduction in number of foci per cell or percentage of cells with foci (Supplementary Fig. S3).

Finally, we evaluated the toxicity of the most active F-ASOs in the NSCs through a colorimetric MTS cell proliferation assay (Supplementary Fig. S5). The 2′F-RNA gapmer ASO4 and its methylated analogue ASO4_Me had no significant effect on cell viability compared to untreated controls. Conversely, the 2′F-ANA gapmer ASO3 reduces cell viability similar to MOE_Ctrl. A more pronounced effect is observed for the 2′F-ANA gapmer ASO3_Me and the 2′F-RNA/LNA gapmer ASO5, showing an ~30% reduction in cell viability, although due to the spread of data points there is no substantial difference between ASO3 and ASO3_Me. Given that ASO5 did not show a significant improvement in activity in either the gene reporter assay or the FISH assay compared to ASO4 and exhibited increased cytotoxicity, this construct was not pursued further in subsequent studies.

### Gymnotic delivery of 2′F-ANA and 2′F-RNA gapmers reduces repeat RNA foci in patient-derived hiPSCs

**Figure 3.**
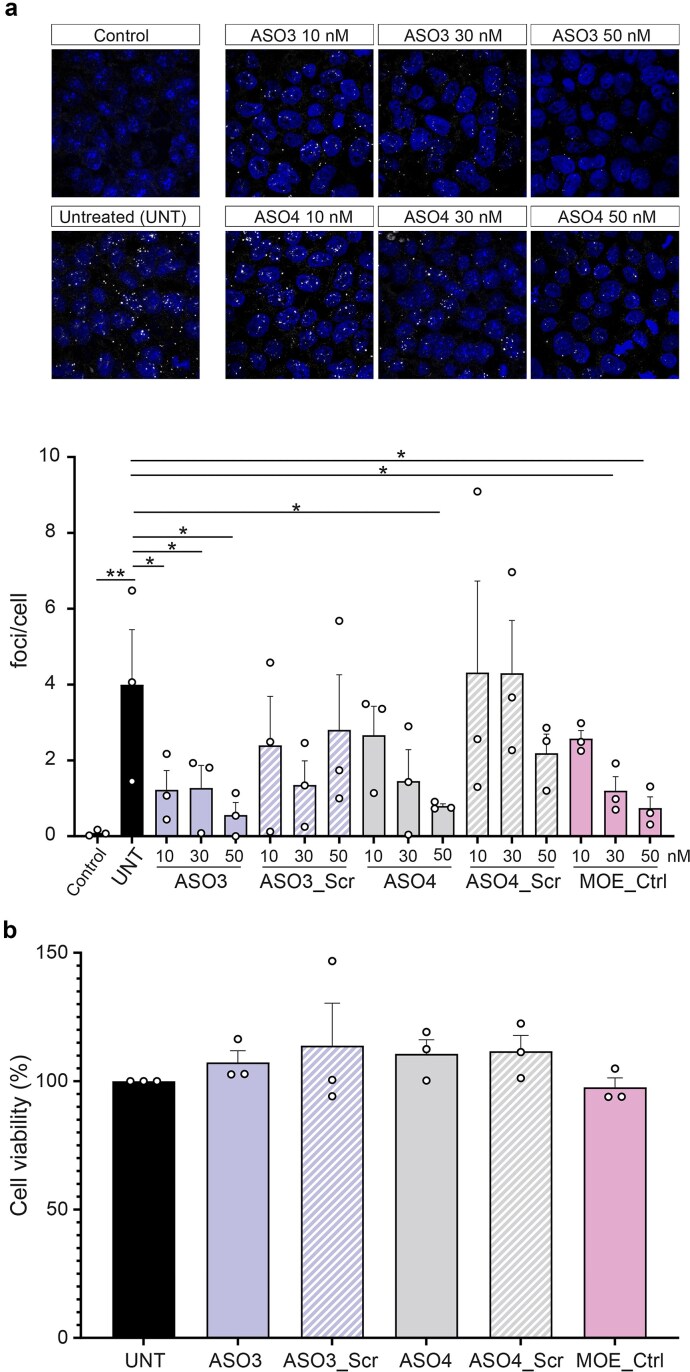
FISH and cell viability assays in patient-derived hiPSCs. (**a**) (Upper panel) Representative confocal microscopy images of healthy control cells, untreated patient-derived cells, and patient-derived cells treated by gymnosis with ASO3 or ASO4 at 10, 30, or 50 nM total ASO concentration (*n* = 3). RNA foci are visualized as bright fluorescent spots detected by FISH and nuclei stained with DAPI. (Lower panel) Quantification of RNA foci per cell following treatment. Bars represent the mean ± SEM from three independent experiments. Statistical significance was determined using one-way ANOVA followed by LSD multiple comparisons. *P-value <.05; **P-value <.01. (**b**) Mean percentage of cell viability determined by an MTS assay following treatment with each ASO (50 nM total ASO concentration, *n* = 3), normalized to untreated cells. Values and error bars represent the mean ± SEM relative to untreated cells from three independent experiments. Statistical significance was determined using one-way ANOVA with LSD correction. No statistically significant differences in cell viability were observed.

Based on our results, ASO3 and ASO4 were selected as the most promising F-ASOs for further characterization in hiPSCs derived from a patient with ALS. Both F-ASOs, their scrambled controls, and MOE_Ctrl were administered by gymnosis (i.e. without transfection agents) at concentrations of 10, 30, and 50 nM. ASO3 significantly reduced RNA foci per cell at the three concentrations tested, indicating strong potency at low doses. ASO4 also produced a significant reduction of RNA foci per cell at 50 nM, whereas MOE_Ctrl showed a significant reduction in RNA foci at 30 nM and 50 nM (Fig. 3a). Similar to the FISH data in the NSCs, both scrambled controls exhibit sporadic effects, although these results are non-significant except for treatment with 30 nM ASO3_Scr, with reduction in foci approaching significance. This sporadic reduction in foci may be linked to the partial complementarity between these GC-rich sequences and the chemistries employed.

To exclude potential cytotoxic effects under the conditions used for these FISH experiments, cell viability was evaluated in the patient-derived hiPSCs using an MTS assay, as performed for the NSCs. No significant reduction in cell viability was observed for ASO3 or ASO4 at 50 nM total ASO concentration, corresponding to the highest dose used in the FISH assays (Fig. 3b). Likewise, no cytotoxic effects were detected for the scrambled controls (ASO3_Scr and ASO4_Scr) or for the MOE_Ctrl under the same conditions (Fig. 3b). These results indicate that the reductions in RNA foci observed in hiPSCs are not attributable to compromised cell viability.

### Active F-ASOs self-fold into secondary structures

The high GC content and repetitive nature of the ASO sequences make them prone to adopt secondary structures such as duplexes, hairpins, G-quadruplexes, or i-motifs. Additionally, the presence of fluorine at the 2′ position of the sugar may stabilize these adopted conformations [48–54]. Self-folding of the ASOs may have consequences on their efficiency as antisense agents. Therefore, we explored whether the ASOs were structured *in vitro*. Firstly, we acquired 1D ^1^H-NMR spectra of the oligonucleotides at 20 and 37°C in a buffer mimicking the salt content considered present in the intracellular environment [55]. The imino region of the NMR spectra of the 24-mer PS full 2′F-ANA (ASO1) shows barely detectable imino signals between 12 and 14 ppm at 20 and 37°C (Supplementary Fig. S6), which indicates that the oligonucleotide remains mostly unfolded under these conditions. Its PO analogue ASO1_PO and the 2′F-ANA/PO gapmer ASO2_PO show a few sharper and more intense imino signals that mostly disappear at 37°C (Supplementary Fig. S6). Interestingly, the spectrum of the 24-mer 2′F-ANA gapmer ASO2 at 20°C shows broad signals at around 15.5 and 13 ppm, characteristic of C:C^+^ and canonical G:C base pairs, respectively.

**Figure 4.**
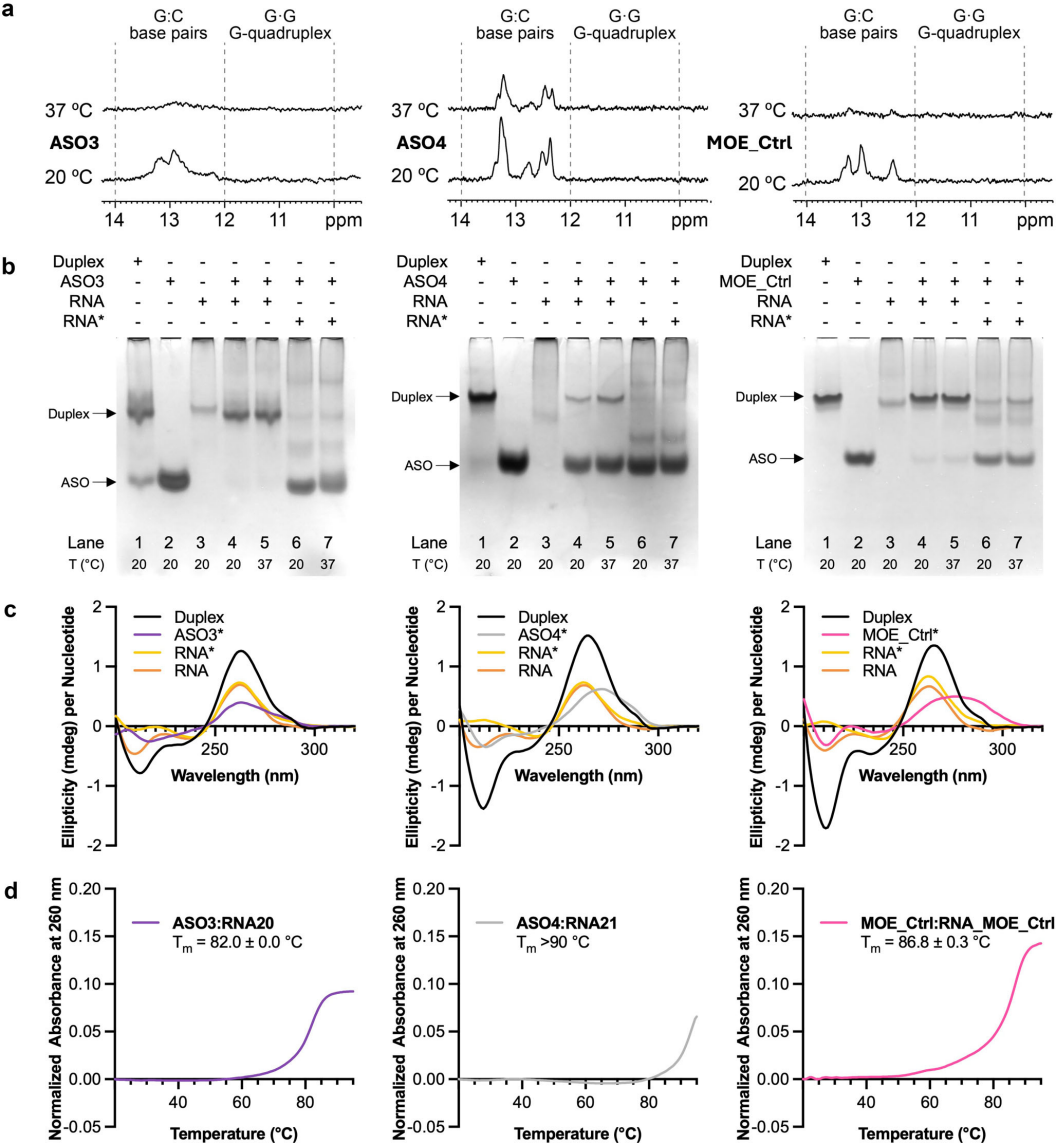
**a**) Imino proton region of the ^1^H NMR spectra of ASO3, ASO4, and MOE_Ctrl at 20 and 37°C. Signals between 12 and 14 ppm correspond to canonical G:C base pairing. (**b**) Native PAGE showing the migration pattern of: ASO:RNA duplex formed upon mixing and slow annealing (lane 1), ASO (lane 2), complementary RNA (lane 3), overnight incubation of the ASO and non-annealed complementary RNA at 20°C (lane 4) and 37°C (lane 5), and overnight incubation of the ASO and annealed complementary RNA (RNA*) at 20°C (lane 6) and 37°C (lane 7). (**c**) CD spectra of the ASO:RNA duplexes, the annealed ASO (ASO*), the annealed RNA (RNA*), and the non-annealed RNA (RNA). (**d**) UV melting curves and melting temperature (T_m_) values of ASO:RNA duplexes.

The spectrum of the shorter 2′F-ANA gapmer ASO3 at 20°C shows signals between 12 and 14 ppm, characteristic of the imino protons involved in canonical G:C base pairs (Fig. 4a). These signals are no longer detected at physiological temperature, indicating that this 2′F-ANA oligonucleotide is unfolded at such temperature. The same is observed for MOE_Ctrl. On the contrary, the imino signals observed in the spectra of the 2′F-RNA gapmer ASO4 remain visible at 37°C, indicating that the structure formed is stable at physiological temperature. In all cases, the broad linewidth likely reflects the presence of multiple diastereomeric species associated with phosphorothioate chirality.

**Figure 5.**
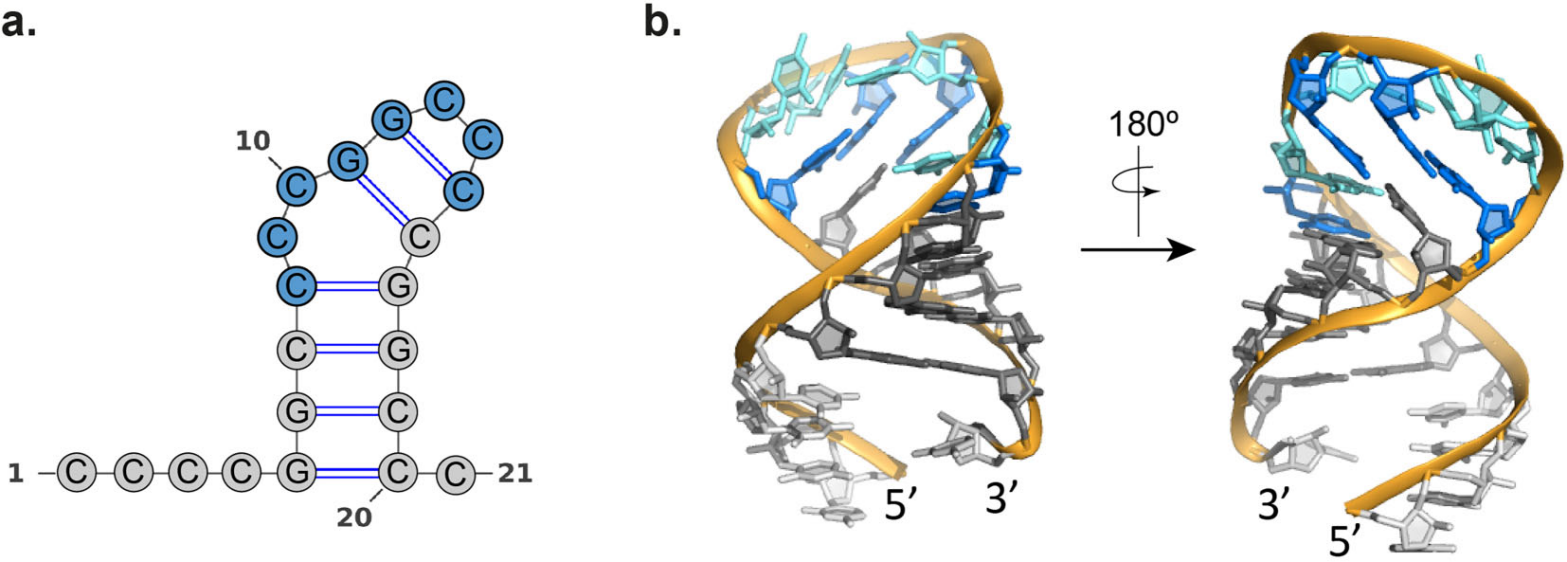
(**a**) Proposed secondary structure based on NMR data. (**b**) Two views of the 3D model of ASO4_PO built with the program Vfold RNA 3D. Color code: dark gray, base-paired 2′F-RNA nucleotides; light gray, unpaired 2′F-RNA nucleotides; blue, base-paired DNA nucleotides; cyan, unpaired DNA nucleotides.

To address this issue, we prepared a pure phosphate variant of ASO4 (ASO4_PO). The NMR spectra of this construct showed narrower and more well-defined signals (Supplementary Fig. S7). Conformational heterogeneity is significantly reduced, as evidenced by the number of H5-H6 cross-peaks observed in the TOCSY spectra (Supplementary Fig. S7), which corresponds to the number of cytosines in the sequence. The high quality of the NMR spectra allows us to confirm the presence of six canonical G:C base pairs. Based on this experimental evidence, we propose a plausible structural model for ASO4 (Fig. 5), consisting of a bulged hairpin structure with a tight loop and an additional helical bulge formed by DNA cytosines. The stem comprises a short A-form helix made up of either 2′F-RNA:2′F-RNA or hybrid 2′F-RNA:DNA G:C base pairs. The well-established stabilizing effect of base pairs containing 2′F-RNA nucleotides contributes significantly to the overall stability of this structure [56]. Consistent with this, UV melting experiments showed that ASO4 has a high melting temperature (T_m_) of 75.5°C, very similar to its complementary G-rich RNA (RNA21) (Supplementary Fig. S8), further supporting the remarkable thermal stability of the proposed structure.

The target RNAs of these ASOs contain the G_4_C_2_ repeat. As already reported for similar RNAs, these oligonucleotides are prone to fold into G-quadruplexes and hairpins under different conditions [57–63]. We analyzed two target RNA fragments, the 20-mer complementary to ASO3 (RNA20) and the 21-mer complementary to ASO4 (RNA21), by NMR under the same conditions indicated above. The spectra of both RNAs show broad imino signals between 12 and 14 ppm and between 10 and 12 ppm (Supplementary Fig. S9), characteristic of protons involved in G:C base pairs and in G-tetrads present in G-quadruplexes, respectively. This suggests a structural scenario where duplex and hairpin structures coexist with G-quadruplexes at 20°C as observed in previous studies of similar RNAs [60, 61, 64]. At higher temperatures, the signals corresponding to G-tetrads keep approximately the same intensity, whereas the signals from G:C base pairs reduce their intensity or even disappear, indicating that G-quadruplex species are more stable at physiological temperature. Consequently, the melting transitions for RNA21 shown in Supplementary Fig. S8 represent apparent, ensemble-averaged T_m_ values. Notably, the absence of an inverted transition at 295 nm for this RNA further supports a heterogeneous ensemble of coexisting conformations rather than a single dominant G-quadruplex [65].

### Folding of F-ASOs into mismatched hairpins does not prevent hybridization with target RNA

Competition between stable self-folding of ASOs or RNA targets and the subsequent formation of ASO:RNA duplexes determines the success of functional hybridization. We used native gel electrophoresis to determine if folded ASOs are able to hybridize to their complementary RNAs *in vitro* under physiological temperature, salt, and pH conditions.

To determine where the ASO:RNA duplex migrates in the gel during electrophoresis, the two oligonucleotides were mixed and annealed (lane 1) (Fig. 4b). Under these conditions, a major slow-migrating band corresponding to the ASO:RNA duplex is observed, with a minor fast-migrating band observed corresponding to unbound ASO. Considering the NMR data (Fig. 4a) and their faster migration with respect to the ASO:RNA duplexes, we infer that the three ASOs (lane 2) are either unstructured or hairpins with C·C mismatches. This is consistent with the circular dichroism (CD) spectra for ASO4 and MOE_Ctrl, showing a positive peak between 260 and 280 nm and a negative peak near 210 nm (Fig. 4c), which is characteristic of A-like duplex character (Supplementary Fig. S10). In contrast, CD of ASO3 at RT shows a shape compatible with some population of the oligonucleotide forming a hairpin with partial B-like character, as expected for a stem containing 2′F-ANA:2′F-ANA base pairs [52] (Fig. 4c). The third lane shows the migration pattern of the target RNA, consisting of a fast-migrating thin band, a band at the top of the well, and a streak connecting the two. This is consistent with the well-documented tendency of r(G_4_C_2_)_n_ to form aggregates [61, 66–68], some of which limit their mobility in the gel matrix. The fast-migrating band migrates to a similar retention as the ASO:RNA duplex, indicating that an RNA duplex with G·G mismatches dominates over the unimolecular hairpin.

For ASO3, upon incubation at 20 or 37°C (lanes 4 and 5), the ASO3:RNA duplex band shows similar intensity. Interestingly, the band corresponding to unbound ASO3 is not observed, suggesting complete hybridization (Fig. 4b). In the case of ASO4, the ASO4:RNA duplex band is also clearly visible at both temperatures, with slightly greater intensity at 37°C incubation (Fig. 4b). For this ASO, the band corresponding to unimolecular structures remains visible, suggesting that a notable population of the oligonucleotide remains self-folded instead of bound to the complementary RNA. The same experiment was performed for MOE_Ctrl, which, according to NMR data, is mostly unfolded in these conditions. The majority of MOE_Ctrl forms the duplex with its complementary RNA upon incubation at both temperatures (Fig. 4b). Control experiments using the scrambled sequences ASO3_Scr and ASO4_Scr show no duplex formation regardless of mixing conditions (Supplementary Fig. S11).

Finally, we explored whether the ASOs are able to form the duplex when the target RNA has been previously annealed. As observed in native PAGE, annealing of the complementary RNAs RNA20 and RNA21 leads to a different migration pattern and, consequently, a different structural scenario (Supplementary Fig. S12). Annealing also affects the shape of the CD spectra of the complementary RNAs. While the non-annealed RNA samples show the characteristic signature of an A-type helix with maximum peak at 265 nm and a minimum at 210 nm, the spectra of the annealed RNAs do not show the minimum peak at 210 nm, instead showing only maximum at 265 nm (Fig. 4c). This indicates that the annealing promotes the unfolding of the A-form duplex and the formation of other structures such as parallel G-quadruplexes. When we incubated equimolar mixes of ASO and annealed RNA at 20 and 37°C (lanes 6 and 7), the ASO:RNA duplex was detected for ASO3 and MOE_Ctrl as a band of lower intensity compared to the one detected when RNA was not annealed (Fig. 4c). This suggests that the annealed RNA is less accessible to the ASO than the non-annealed RNA. In the case of ASO4, only a barely detectable band is observed. Unsurprisingly, ASO3_Scr and ASO4_Scr do not bind the annealed RNA (Supplementary Fig. S11).

### Hybridization of F-ASOs and their target RNA forms very stable A-like duplexes

The structural features of the ASO:RNA duplex determine the mechanism through which gene expression is affected. We used CD to determine the conformation of the duplex resulting from hybridization of the ASOs with their target RNAs. To compare CD spectra of duplexes with different numbers of base pairs, we plotted ellipticity per nucleotide. CD spectra of the ASO3:RNA20 and ASO4:RNA21 duplexes at RT revealed maxima at around 265 nm and minima at 210 nm (Fig. 4c), which are characteristic of A-like duplex conformation [69]. However, whereas the intensity of the peak at 265 nm is very similar in all cases, the peak at 210 nm shows variable intensity depending on the duplex. The intensity of the peak at 210 nm is indicative of how similar the duplex is to a pure A-form conformation given by RNA:RNA double helix, which shows an intense negative peak (Supplementary Fig. S10). Accordingly, the ASO:RNA duplexes involving the 2′F-RNA gapmer ASO4 and MOE_Ctrl have notably more A-form character than the 2′F-ANA gapmer ASO3.

We also evaluated the thermal stability of the ASO:RNA duplexes by UV-monitored thermal melting assays (Fig. 4d and Supplementary Table S1). The melting temperature of the ASO3:RNA duplex is ~82°C, while this value exceeds 90°C for ASO4:RNA, even at low salt concentrations where the duplex is less stable. The duplex formed by MOE_Ctrl and its complementary RNA has a melting temperature of ~87°C, and therefore its thermal stability is between that of the 2′F-ANA:RNA duplex and the 2′F-RNA:RNA duplex.

## Discussion

In this study, we synthesized and evaluated a series of fluorine-modified ASOs targeting the 5′-GGGGCC-3′ repeats associated with *C9ORF72* repeat expansion mutations, a major genetic cause of ALS and FTD. While our initial screening included additional designs, we chose to focus our study on a representative subset to ensure a cohesive narrative across our biochemical, biophysical, and cellular analyses. Our primary objective was not to systematically optimize ASO architecture, but to demonstrate that F-ASOs prone to self-folding can effectively target G-rich repeat RNAs, such as those from the *C9ORF72* gene, and maintain their functionality in cellular systems.

Cell-based screening confirmed that a phosphorothioate (PS) backbone is required for cellular activity of F-ASOs, consistent with prior reports showing that PS modification enhances nuclease resistance, cellular uptake, and RNase H recruitment relative to phosphodiester (PO) backbones [70, 71]. In addition, comparison across designs indicates that gapmer architectures are more effective than fully modified ASOs in both the reporter cells and patient-derived NSC FISH assays, whereas ASO length has only a minor influence on activity, with shorter constructs showing comparable or slightly improved performance.

Based on the results obtained from reporter gene expression and RNA foci analyses in NSCs, three F-ASOs (ASO3, ASO4, and ASO5) showed the highest activity among the designs evaluated. Among these candidates, the 2′F-RNA gapmer ASO4 did not affect NSC viability, the 2′F-ANA gapmer ASO3 showed only modest toxicity comparable to MOE_Ctrl, and the 2′F-RNA gapmer ASO5, which incorporates LNA residues, exhibited increased cytotoxicity. Such increased cytotoxicity is consistent with prior reports linking LNA modifications to hepatotoxicity and RNase H-mediated off-target effects, which are known to depend on both sequence composition and the precise positioning of LNA residues within gapmer architectures [72–77]. Given its activity in the selection assays, these results suggest that improved LNA/2′F-RNA designs could be achieved through optimized placement and chemistry to reduce cytotoxicity.

Treatment of patient-derived hiPSCs carrying the *C9ORF72* repeat expansion with ASO3 or ASO4, administered by gymnotic delivery, resulted in a significant reduction in the number of nuclear RNA foci. Notably, the 2′F-ANA gapmer ASO3 produced a significant effect at concentrations as low as 10 nM, indicating high potency under gymnotic conditions and exceeding the efficacy of a gapmer control bearing the widely used 2′-MOE modification, which showed significant activity only at 30 nM. The 2′F-RNA gapmer ASO4 also reduced RNA foci, although a higher concentration of 50 nM was required to achieve statistical significance. These results suggest a lower efficiency or poorer uptake of ASO4 compared to ASO3. Since 2′F-ANA is known to enhance gymnotic uptake of oligonucleotides [35], it is likely that ASO3 enters cells more efficiently. Nonetheless, the ability to achieve significant foci reduction at nanomolar concentrations is notable, as these doses are substantially lower than those typically reported for ASOs administered by gymnotic delivery in comparable cellular models [78–80]. Together with the results obtained in the earlier selection assays, these findings demonstrate that fluorinated chemistries can achieve levels of activity comparable to established antisense chemistries in patient-derived cells using gymnotic delivery without compromising cell viability at the concentrations tested.

Exploring the structure of an ASO and its target RNA is particularly relevant when the nucleic acids intended for hybridization have the intrinsic ability to form stable secondary structures. The formation of stable structures by the target and the interacting nucleic acid may compromise the formation of the ASO:RNA duplex required to activate antisense mechanisms. On the other hand, the folding adopted by the ASO may have a positive impact on its activity as it could improve nuclease resistance or cellular uptake. Our structural analysis reveals that the 2′F-RNA-modified gapmer ASO4 adopts a stable folded structure at 37°C in a near-physiological buffer, suggesting that it is likely to retain this conformation upon administration in cell-based assays and potentially also in animals. Based on NMR and native PAGE results, we attribute this structure to a mismatched hairpin stabilized by G:C base pairs. This structured ASO exhibits activity comparable to ASO3 and MOE_Ctrl, which adopt secondary structures only at lower temperatures, in cell-based assays. Indeed, the self-folding of ASO4 does not substantially preclude their binding to their complementary RNA sequences, as demonstrated by native PAGE. Consistently, the melting temperature of the ASO4:RNA duplex (T_m_ > 90°C) is substantially higher than that of the self-folded ASO4 alone (T_m_ ∼75°C), indicating a clear energetic preference for target binding over self-association. Nevertheless, intrinsic self-folding propensity correlates with both target-binding efficiency *in vitro* and RNA foci reduction in hiPSCs. Native PAGE shows that ASO3 and MOE_Ctrl form a larger fraction of ASO:RNA duplex compared to ASO4. Moreover, ASO3 and MOE_Ctrl retain the ability to bind their complementary RNAs, albeit to a much lower extent, under annealing conditions in which heating and slow cooling conditions that favor RNA self-association into multiple conformations, including G-quadruplexes and multimeric species.

In line with these observations, the 2′F-ANA gapmer ASO3 shows significant RNA foci reduction in hiPSCs at the lowest concentration tested, followed by MOE_Ctrl, whereas the more structured 2′F-RNA gapmer ASO4 requires higher concentrations to achieve a comparable effect. In contrast, scrambled controls ASO3_Scr and ASO4_Scr failed to significantly reduce RNA foci at any concentration. These sequences were designed to match the chemistry and GC-rich composition of ASO3 and ASO4. Although low-affinity cellular interactions cannot be fully ruled out given the repeat-containing RNA target, these controls demonstrated no activity in the reporter cells or *in vitro* binding assays. These results confirm that the biological effects of ASO3 and ASO4 are sequence-specific and driven by an antisense mechanism.

Finally, CD spectroscopy indicates that the ASO:RNA duplexes formed by ASO3, ASO4, and MOE_Ctrl adopt an A-like helical conformation. This, together with the gapmer architecture of the active F-ASOs, is consistent with RNase H-mediated cleavage of the target RNA, as reported for related 2′F-ANA/PS gapmer chemistries [34].

A key strength of targeting the expanded *C9ORF72* repeat with ASOs lies in the high density of binding sites provided by the repetitive RNA sequence, which may enhance local ASO occupancy and overall antisense activity [23, 24]. Notably, ASOs that advanced into clinical trials, such as BIIB078 (tadnersen) and WVE-004, were designed to reduce repeat-containing transcripts without directly targeting the G_4_C_2_ repeat region and were ultimately discontinued due to lack of clinical benefit [28, 29]. In this context, directly targeting the repeat sequence itself may offer an alternative strategy with a potentially different functional outcome compared to approaches that act outside the repeat region. In addition, our data show that the use of fluorinated ASOs, particularly those incorporating 2′F-ANA, combines antisense competence with significant potency under gymnotic delivery conditions at low nanomolar concentrations [33–35, 81, 82]. On the other hand, this strategy also presents inherent challenges, such as the tendency of both the repeat region of the RNA and the ASOs to form stable structures, which our data show may impact the efficiency of ASO:RNA duplex formation. More broadly, while 2′F-ANA is a well-characterized and potent modification, potential liabilities associated with antisense therapies, such as off-target effects, sequence-dependent toxicity, and chemistry-specific safety concerns, remain to be fully addressed.

Our findings support the use of F-ASOs as effective tools for targeting repeat RNAs in contexts such as those associated with C9FTD/ALS and other repeat expansion diseases, where both the ASO and the target RNA may adopt stable secondary structures. Nonetheless, we acknowledge that *in vivo* validation will be essential to fully assess their translational potential. In this regard, the methylated variants of ASO3 and ASO4, which were designed to mitigate potential CpG-mediated toxicity without affecting activity, represent particularly attractive candidates for *in vivo* evaluation. Future studies incorporating complementary readouts such as target mRNA and protein quantification, as well as systematic evaluation of ASO architecture, will help refine the design of F-ASOs.

## Supplementary Material

gkag343_Supplemental_File

## Data Availability

The data underlying this article are available in the article and in its online supplementary material.
