# Supplementary material for: Direct targeting of C9ORF72 repeat RNA with fluorinated antisense oligonucleotides
Source: Nucleic Acids Res. 2026 Apr 25;54(8):gkag343. doi: 10.1093/nar/gkag343 (PMC13109722; doi:10.1093/nar/gkag343)
Supplement: gkag343_Supplemental_File [file gkag343_supplemental_file.pdf]

Supplementary Data

Direct targeting of *C9ORF72* repeat RNA with fluorinated antisense oligonucleotides

AUTHORS

Halle M. Barber[1,†], Mansi A. Parasrampuria[2,†], Jerónimo Jurado-Arjona[4,5,†], Andrea Gamir-Morralla[4,5], Benedikt Berninger[4,5,6,7], Carlos González[8], Keith T. Gagnon[2,3,*], Masad J. Damha[1,*], and Miguel Garavís[8,*]

[1] Department of Chemistry, McGill University, Montreal, QC, H3A 0B8, Canada

[2] Biochemistry and Molecular Biology, Wake Forest University School of Medicine, Winston-Salem, NC, 27101, USA

[3] Division of Biochemistry and Molecular Biology, Southern Illinois University School of Medicine, Carbondale, IL, 62901, USA

[4] Centre for Developmental Neurobiology, Institute of Psychiatry, Psychology & Neuroscience, King's College London, London, WC2R 2LS, UK

[5] Institute of Physiological Chemistry, University Medical Center Johannes Gutenberg University, Mainz, 55131, Germany

[6] MRC Centre for Neurodevelopmental Disorders, Institute of Psychiatry, Psychology & Neuroscience, King's College London, London, WC2R 2LS, UK

[7] Focus Program Translational Neuroscience, Johannes Gutenberg University, Mainz, 55131, Germany

[8] Instituto de Química Física 'Blas Cabrera', IQF-CSIC, Madrid, 28006, Spain

† Halle M. Barber, Mansi A. Parasrampuria, and Jerónimo Jurado-Arjona contributed equally to this work

* To whom correspondence should be addressed. Email: keith.gagnon@advocatehealth.org, masad.damha@mail.mcgill.ca, mgaravis@iqf.csic.es

OUTLINE

Table S1. Oligonucleotide sequences, chemical architectures, and characterization. p2
Figure S1. Representative flow cytometry plots in $(G_4C_2)_1$-mCherry cells. p3
Figure S2. Representative flow cytometry plots in $(G_4C_2)_{88}$-mCherry cells. p4
Figure S3. FISH in patient-derived NSCs. p5
Figure S4. Representative FISH images of patient-derived NSCs. p6
Figure S5. MTS cell viability assay in patient-derived NSCs. p6
Figure S6. 1D ^{1}H-NMR of ASO1_PO, ASO1, ASO2_PO, and ASO2. p7
Figure S7. 1D and 2D ^{1}H-NMR spectra of ASO4_PO. p8
Figure S8. UV melting curves of ASO4 and RNA21. p9
Figure S9. 1D ^{1}H-NMR of complementary RNAs. p9
Figure S10. CD spectra of DNA:RNA, DNA:DNA, and RNA:RNA duplexes. p9
Figure S11. NMR, native PAGE, and CD of ASO3_Scr and ASO4_Scr. p10
Figure S12. Native PAGE of complementary RNAs with and without annealing. p11

Name	Sequence (5′-3′)	Expected Mass	Observed Mass	T_m (ASO:RNA)
ASO1_PO	G-C-C-C-C-G-G-C-C-C-C-G-G-C-C-C-C-G-G-C-C-C-C-G	7626.9906	7626.9688	>90 °C
ASO2_PO	G-C-C-C-C-G-G-C-C-C-C-G-G-C-C-C-C-G-G-C-C-C-C-G	7411.0974	7411.0625	>90 °C
ASO1	G^C^C^C^C^G^G^C^C^C^C^G^G^C^C^C^C^G^G^C^C^C^C^G	7994.9906	7994.4688	>90 °C
ASO2	G^C^C^C^C^G^G^C^C^C^C^G^G^C^C^C^C^G^G^C^C^C^C^G	7779.0974	7778.4688	85.3 ± 0.3 °C
ASO3	C^C^C^C^G^G^C^C^C^C^G^G^C^C^C^C^G^G^C^C	6478.8994	6478.5625	82.0 ± 0.0 °C
ASO3_Me	C^C^C^C^G^G^C^C^C^C^G^G^C^C^C^C^G^G^C^C	6492.9194	6492.5000	*
ASO3_Scr	C^C^G^C^C^G^C^C^G^C^C^C^G^C^C^G^C^C^G^C	6478.8994	6478.5313	**
ASO4	C^C^C^C^G^G^C^C^C^C^G^G^C^C^C^C^G^G^C^C^C	6801.9368	6801.3125	>90 °C (duplex)<br>75.5 ± 0.9 °C (hairpin)
ASO4_Me	C^C^C^C^G^G^C^C^C^C^G^G^C^C^C^C^G^G^C^C^C	6815.9568	6815.4688	*
ASO4_Scr	C^C^G^C^C^G^C^C^G^C^C^C^G^C^C^G^C^C^G^C^C	6801.9368	6801.5000	**
ASO4_PO	C-C-C-C-G-G-C-C-C-C-G-G-C-C-C-C-G-G-C-C-C	6481.9368	6481.8382	*
ASO5	C^C^C^C^G^G^C^C^C^C^G^G^C^C^C^C^G^G^C^C^C	6898.0172	6897.3828	>90 °C
MOE_Ctrl	C^C^C^G^G^C^C^C^C^G^G^C^C^C^C^G^G^C^C^C	7087.4262	7086.8797	86.8 ± 0.3 °C
DNA_Ctrl	C-C-C-C-G-G-C-C-C-C-G-G-C-C-C-C-G-G-C-C-C	6248.0522	6248.0000	79.0 ± 0.0 °C
RNA_Ctrl	C-C-C-C-G-G-C-C-C-C-G-G-C-C-C-C-G-G-C-C-C	6583.9412	6583.9063	>90 °C
RNA20	G-G-C-C-G-G-G-G-C-C-G-G-G-G-C-C-G-G-G-G	6602.0000	6602.0000	*
RNA21	G-G-G-C-C-G-G-G-G-C-C-G-G-G-G-C-C-G-G-G-G	6947.2000	6947.0000	75.7 ± 0.3 °C
RNA_MOE_Ctrl	G-G-G-C-C-G-G-G-G-C-C-G-G-G-G-C-C-G-G-G	6602.0000	6602.9000	*
DNA21	G-G-G-C-C-G-G-G-G-C-C-G-G-G-G-C-C-G-G-G-G	6611.3000	6610.2000	*

Supplementary Table S1. Sequences, chemical architectures, and molecular weight of the oligonucleotides used in this study. Last column shows the melting temperature (T_m) values of the duplex formed by each ASO with its complementary RNA (* = not measured, ** = no value) and the Tm of the hairpin formed by ASO4. Color code: blue (DNA), purple (2′F-ANA), grey (2′F-RNA), orange (LNA), pink (2′-MOE), and yellow (RNA). Underlined residues are cytosines methylated at position 5. Phosphodiester linkages are indicated by dashes and phosphorothioate linkages are indicated by carets.

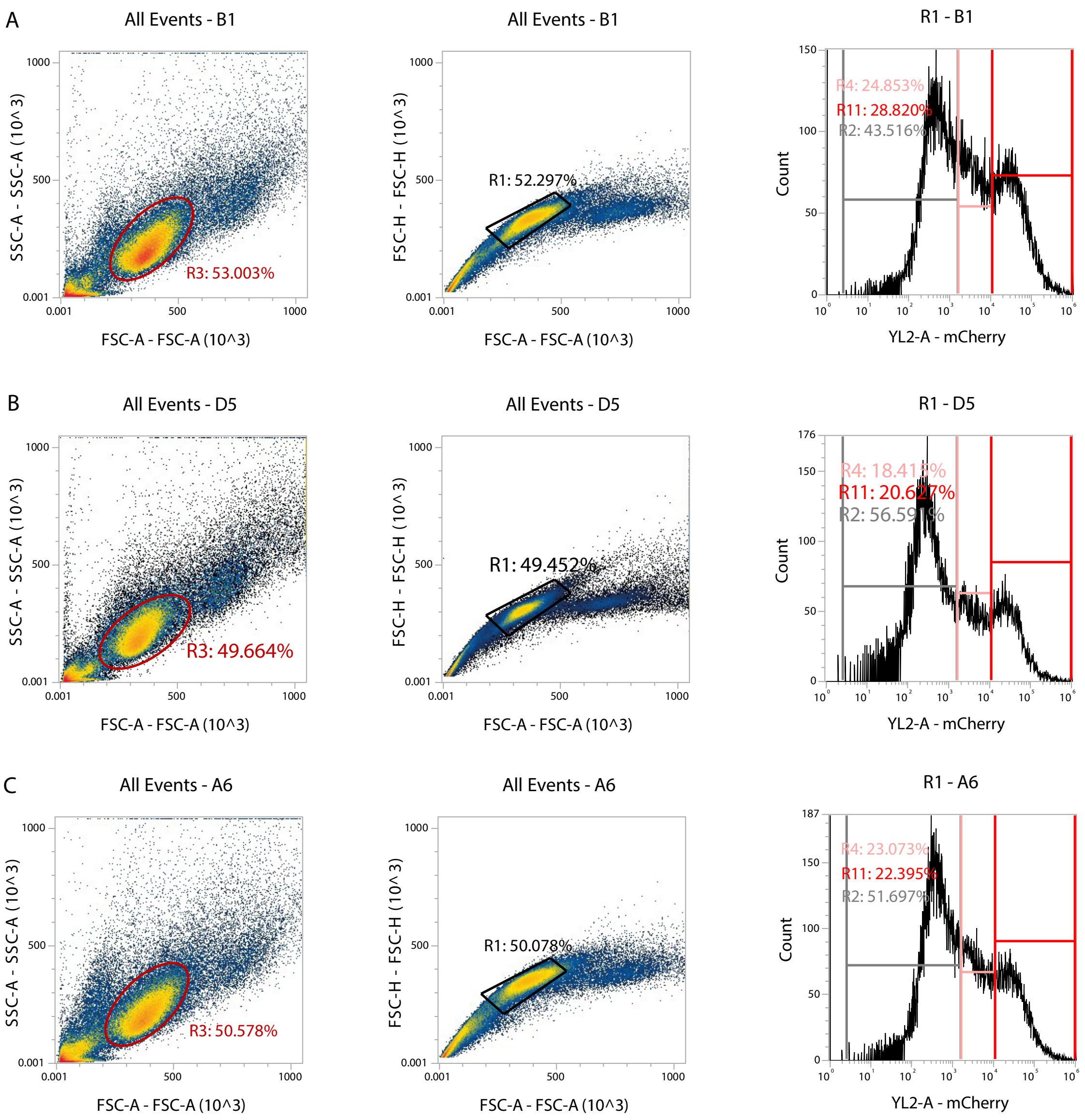

Supplementary Figure S1. Representative flow cytometry plots for (**A**) untreated, (**B**) ASO3, and (**C**) ASO4 in doxycycline-inducible stably expressing $(G_4C_2)_1$-mCherry cells. The flow cytometry was performed 72 h post-ASO transient transfection induced with a doxycycline concentration of 0.5 µg/mL.

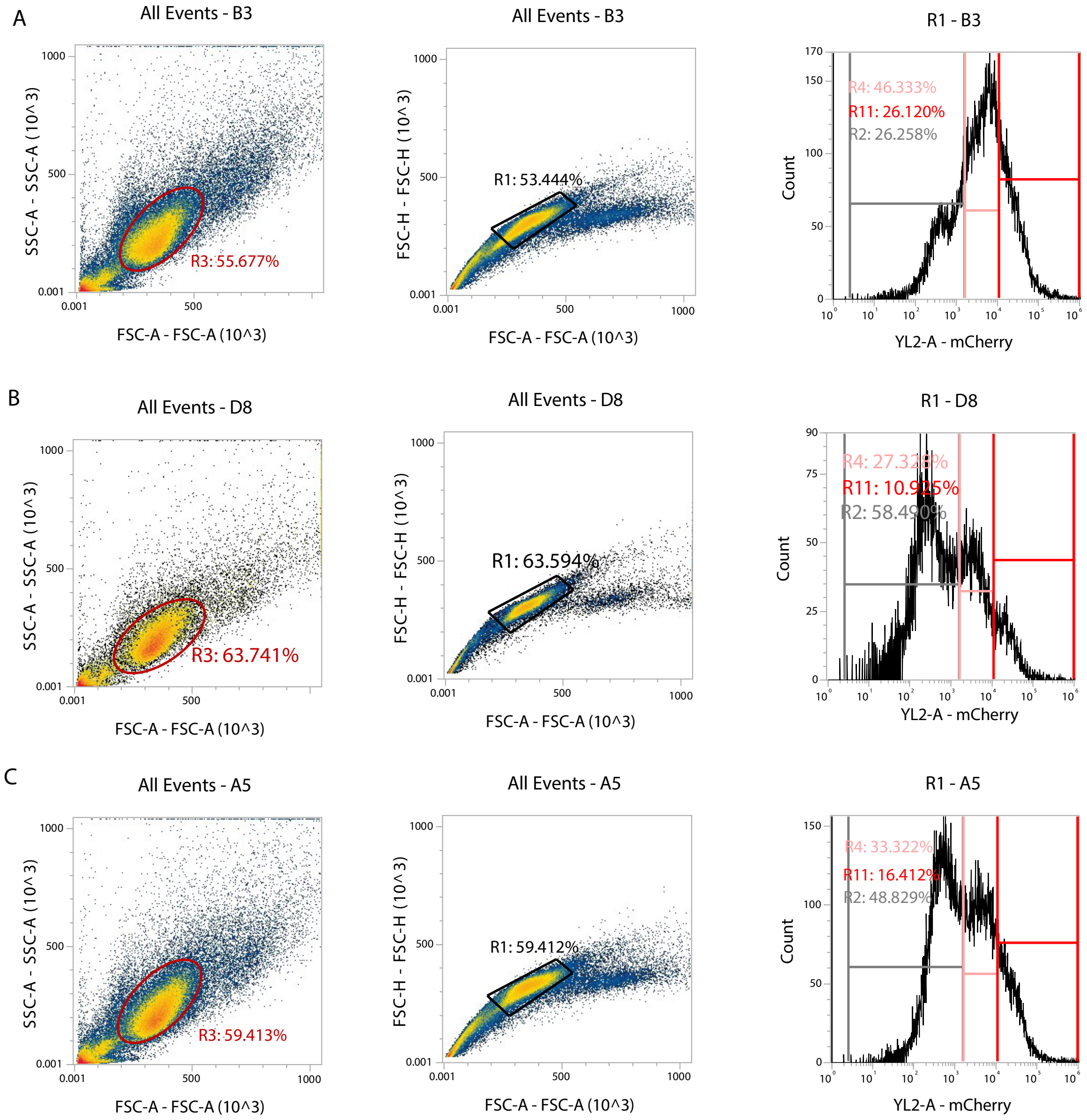

Supplementary Figure S2. Representative flow cytometry for (**A**) untreated, (**B**) ASO3, and (**C**) ASO4 in doxycycline-inducible stably expressing $(G_4C_2)_{88}$-mCherry cells. The flow cytometry was performed 72 h post-ASO transient transfection induced with a doxycycline concentration of 0.5 μg/mL.

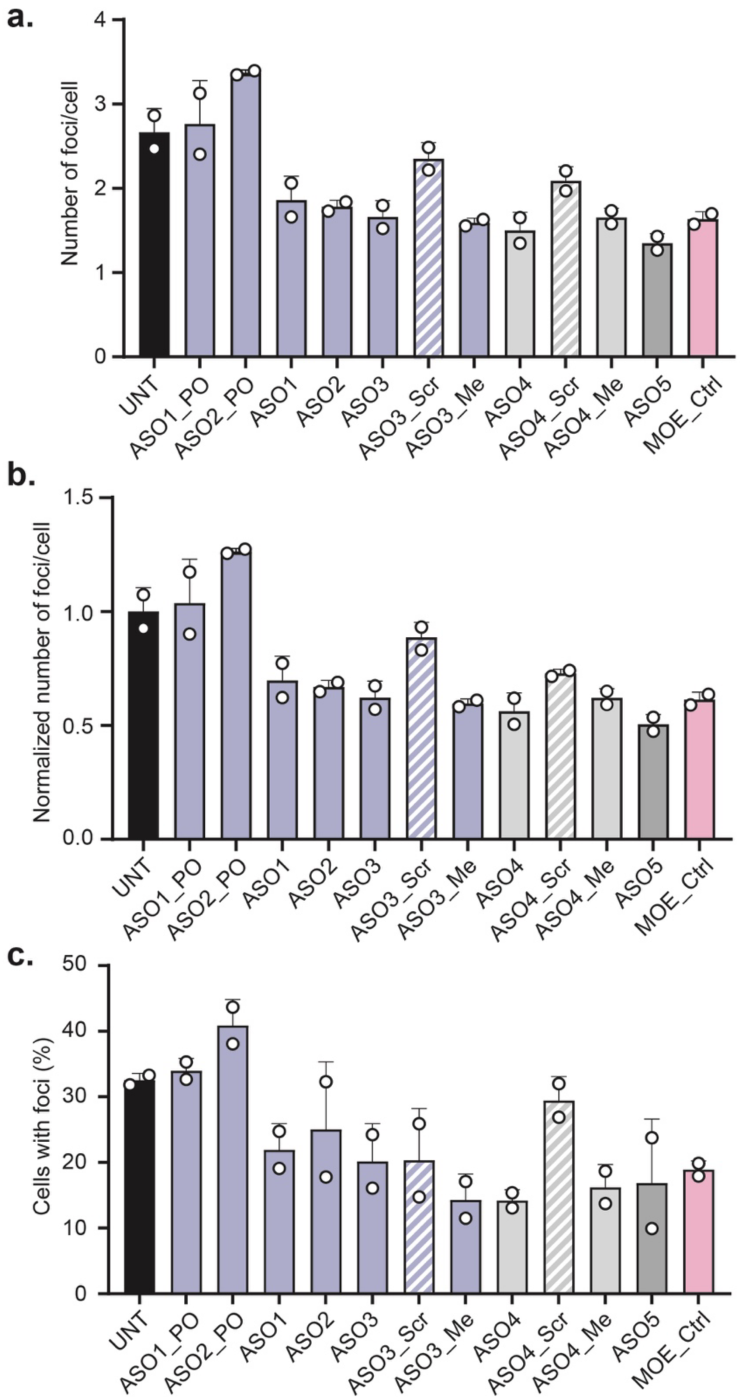

Supplementary Figure S3. Fluorescence *in situ* hybridization (FISH) assay in patient-derived NSCs treated by transfection with 20 nM of total ASO concentration (*n* = 2). **a.** Number of RNA foci per cell. **b.** Number of RNA foci per cell normalized to untreated cells. **c.** Percentage of cells containing RNA foci. Values and error bars represent the mean ± SEM.

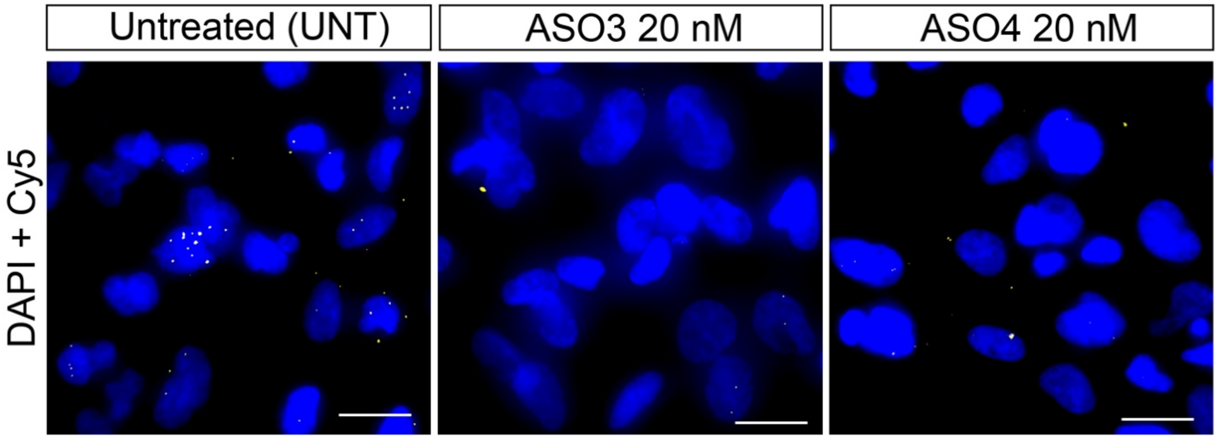

Supplementary Figure S4. Representative FISH images of patient-derived NSCs. Sense RNA foci in yellow and nuclear staining with DAPI in blue. Magnification = 63x; scale bar = 20 µm.

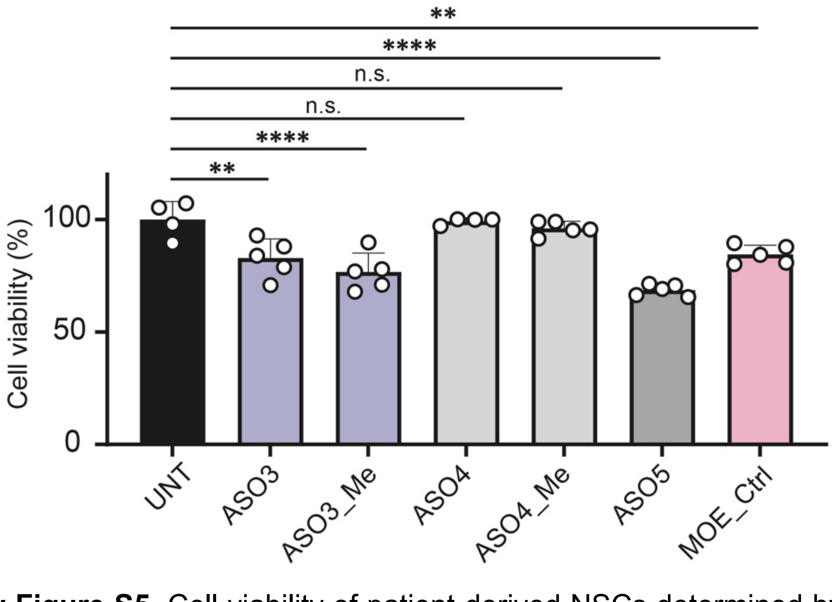

Supplementary Figure S5. Cell viability of patient-derived NSCs determined by an MTS assay at 490 nm upon treatment with 20 nM of total ASO concentration normalized to untreated cells (*n* = 5). Values and error bars represent the mean ± SD. Statistical significance was determined using one-way ANOVA with Dunnett's correction. **p-value < 0.0021; ****p-value < 0.0001; n.s., not significant.

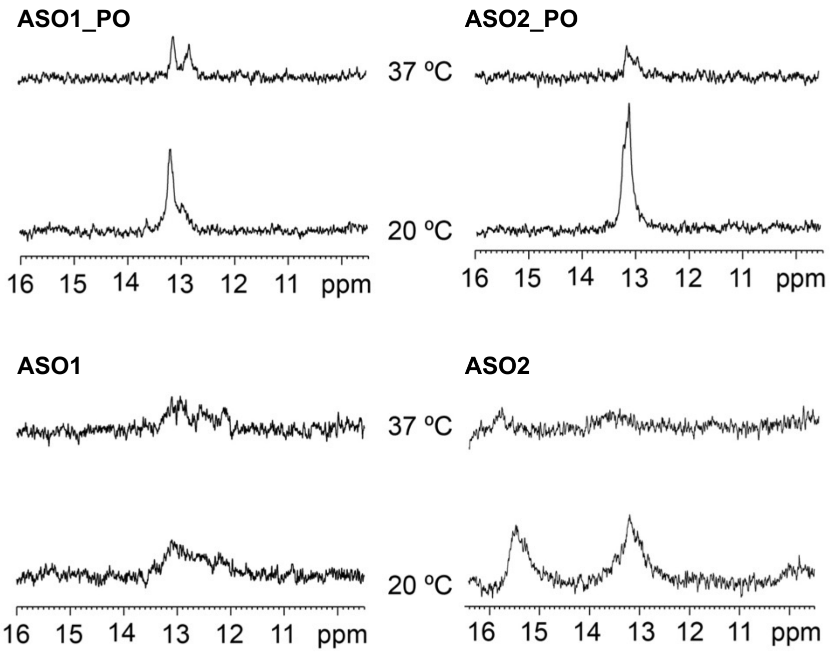

Supplementary Figure S6. Imino region of the 1D ^{1}H-NMR spectra of the 24-mer PO and PS full 2′F-ANA (ASO1_PO and ASO1, respectively) and 24-mer PO and PS 2′F-ANA gapmers (ASO2_PO and ASO2, respectively) at 20 °C and 37 °C. Imino signals at around 15.5 ppm and 13 ppm are characteristic of C:C$^+$ base pairs and canonical G:C base pairs, respectively.

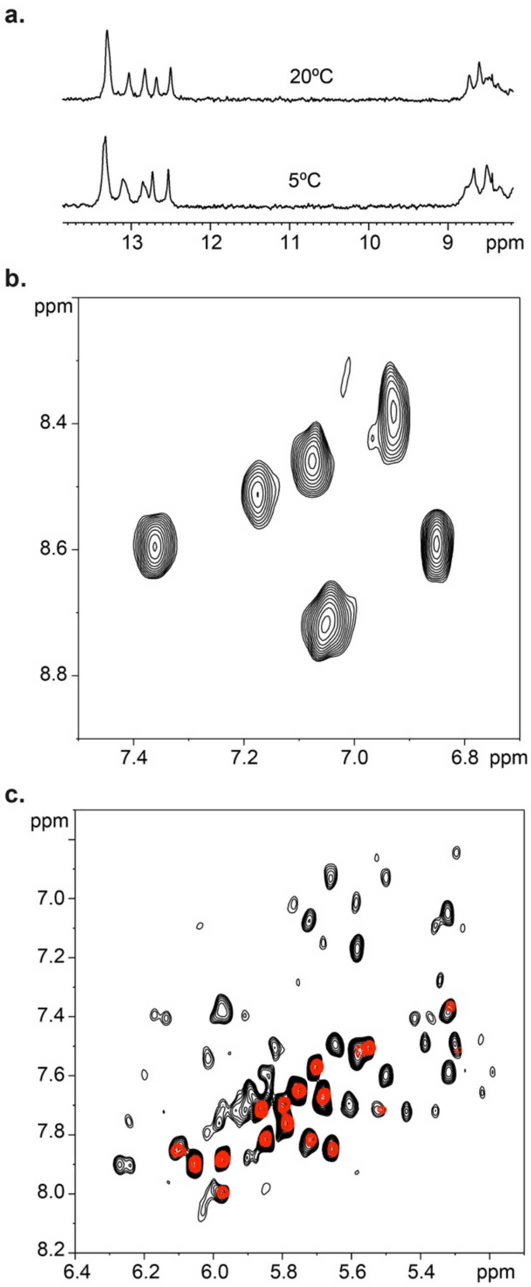

Supplementary Figure S7. **a.** Exchangeable proton spectra of ASO4_PO at 5 and 20 °C. **b.** Region of the NOESY spectrum showing amino-amino NOEs between cytosines (mixing time 150 ms, T = 20 °C). **c.** Superposition of the H1′-aromatic region of the NOESY (black) and TOCSY spectra (red). The number and chemical shifts of guanine imino signals in **(a)** and amino-amino cytosine cross-peaks in **(b)** are consistent with the formation of six canonical G:C base pairs. Moreover, the number of H5-H6 cytosine cross-peaks in **(c)** is consistent with the presence of 15 cytosines, suggesting a well-defined structure.

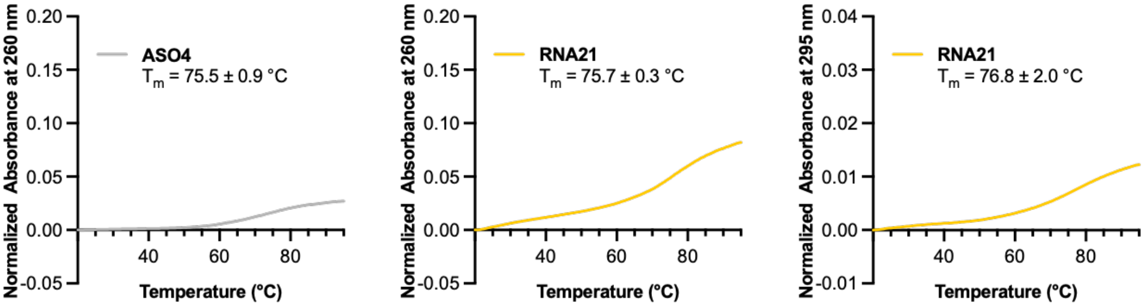

Supplementary Figure S8. UV melting curves and melting temperature (T_m) values of ASO4 and its complementary RNA (RNA21) showing the normalized variation of absorbance at 260 nm and 295 nm (for RNA21) over a range of temperature from 20 to 95 °C.

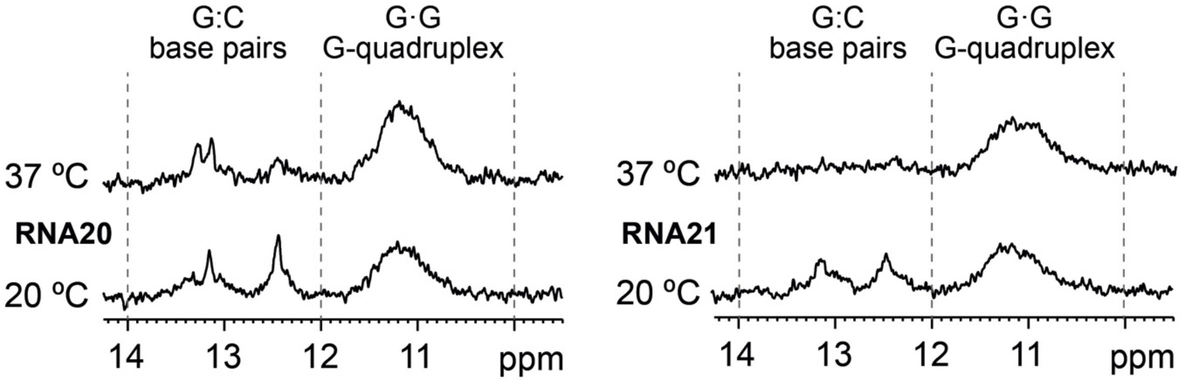

Supplementary Figure S9. Imino proton region of the ^{1}H NMR spectra of RNA20 and RNA21 at 20 °C and 37 °C. Dashed lines highlight spectral regions containing signals characteristic of imino protons involved in canonical G:C base pairs and G-tetrad formation.

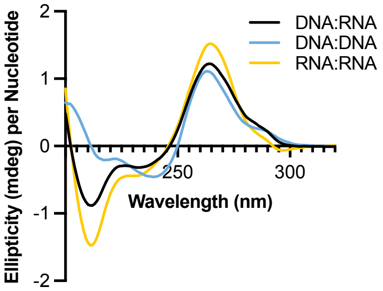

Supplementary Figure S10. CD spectra of unmodified duplexes DNA:RNA (black), DNA:DNA (blue), and RNA:RNA (yellow) using DNA_Ctrl, RNA_Ctrl, and their complementary DNA and RNA strands.

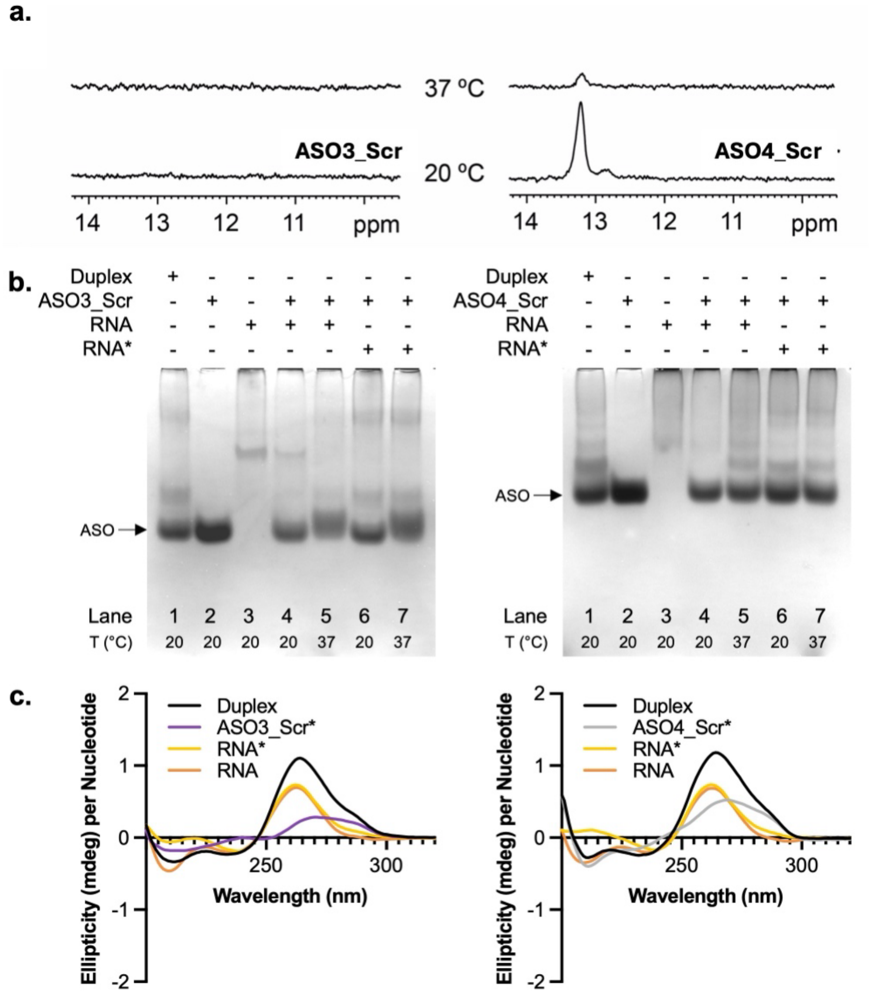

Supplementary Figure S11. a. Imino proton region of the ^{1}H NMR spectra of ASO3_Scr and ASO4_Scr at 20 and 37 °C. Signals between 12 and 14 ppm correspond to canonical G:C base pairing. **b.** Native PAGE showing the migration pattern of: ASO:RNA duplex formed upon mixing and slow-annealing (lane 1), ASO (lane 2), complementary RNA (lane 3), overnight incubation of the ASO and non-annealed complementary RNA at 20 °C (lane 4) and 37 °C (lane 5), and overnight incubation of the ASO and annealed complementary RNA (RNA*) at 20 °C (lane 6) and 37 °C (lane 7). **c.** CD spectra of the ASO:RNA duplexes (black), annealed ASO (ASO*, ASO3_Scr: purple; ASO4_Scr: grey), the annealed RNA (RNA*, yellow), and the non-annealed RNA (RNA, orange).

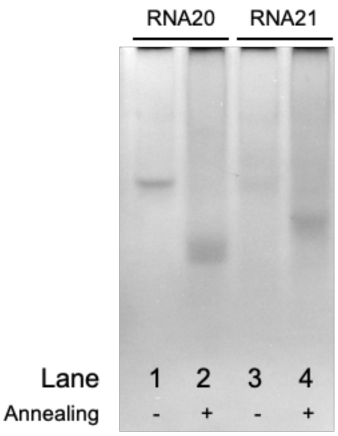

Supplementary Figure S12. Native PAGE of RNA20 and RNA21 either without being subjected to annealing (lanes 1 and 3) or after slow annealing (lanes 2 and 4).
